# Supplementary figures and images for: Host-derived Lactobacillus plantarum alleviates hyperuricemia by improving gut microbial community and hydrolase-mediated degradation of purine nucleosides
Source: eLife. 2024 Nov 7;13:e100068. doi: 10.7554/eLife.100068 (PMC11542919; doi:10.7554/eLife.100068)

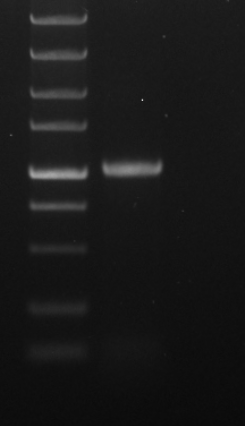

Supplement: Figure 5—figure supplement 1—source data 1. [file elife-100068-fig5-figsupp1-data1.zip › Figure 5—figure supplement 1-source data 1/heterologous expression/LP pcr.tif]

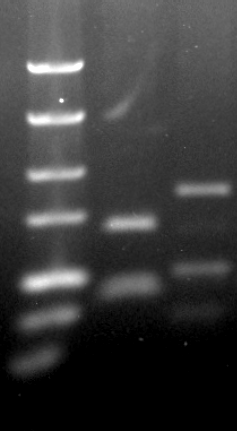

Supplement: Figure 5—figure supplement 1—source data 1. [file elife-100068-fig5-figsupp1-data1.zip › Figure 5—figure supplement 1-source data 1/heterologous expression/p15A-LP-CK-iunH.tif]

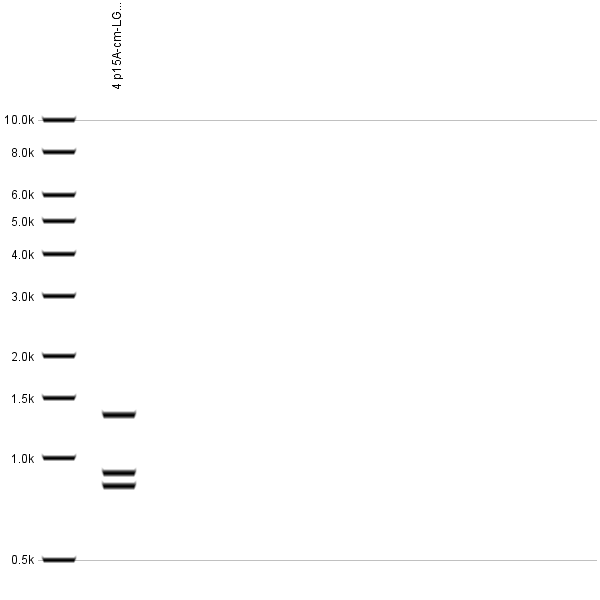

Supplement: Figure 5—figure supplement 1—source data 1. [file elife-100068-fig5-figsupp1-data1.zip › Figure 5—figure supplement 1-source data 1/heterologous expression/p15A-LP-CK.png]

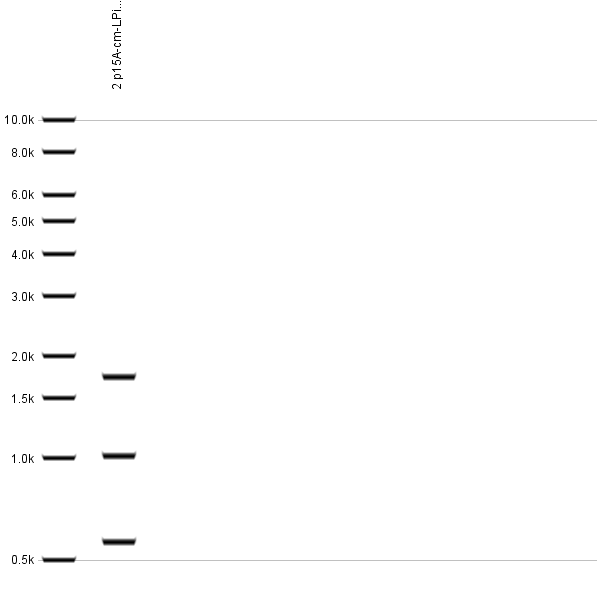

Supplement: Figure 5—figure supplement 1—source data 1. [file elife-100068-fig5-figsupp1-data1.zip › Figure 5—figure supplement 1-source data 1/heterologous expression/p15A-LP-iunH.png]

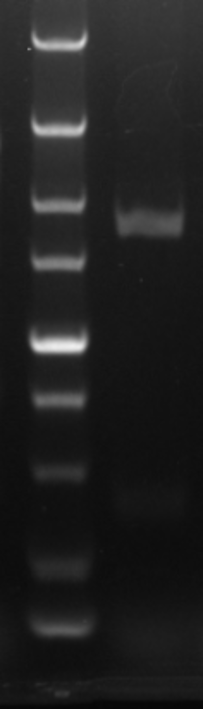

Supplement: Figure 5—figure supplement 1—source data 1. [file elife-100068-fig5-figsupp1-data1.zip › Figure 5—figure supplement 1-source data 1/knockout (gene)/LP pcr.tif]

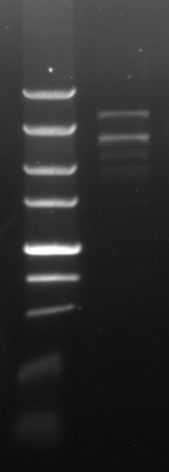

Supplement: Figure 5—figure supplement 1—source data 1. [file elife-100068-fig5-figsupp1-data1.zip › Figure 5—figure supplement 1-source data 1/knockout (gene)/p15A-scaB-iunH.tif]

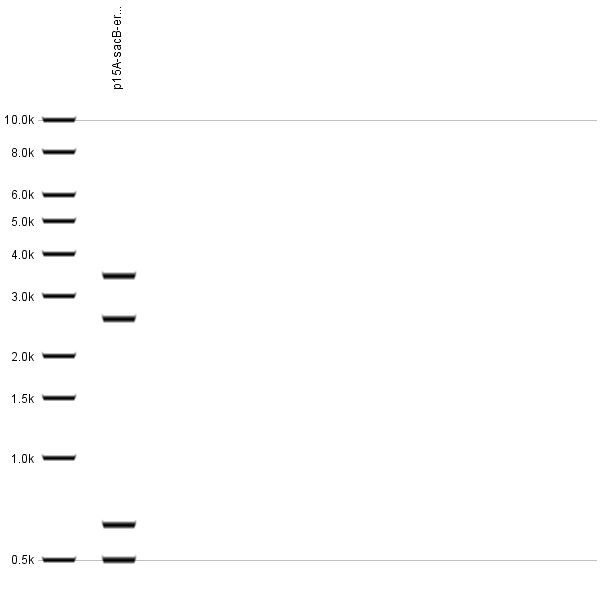

Supplement: Figure 5—figure supplement 1—source data 1. [file elife-100068-fig5-figsupp1-data1.zip › Figure 5—figure supplement 1-source data 1/knockout (gene)/scaB-iunH.png]

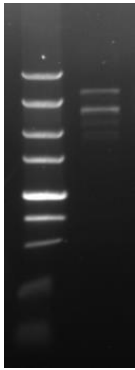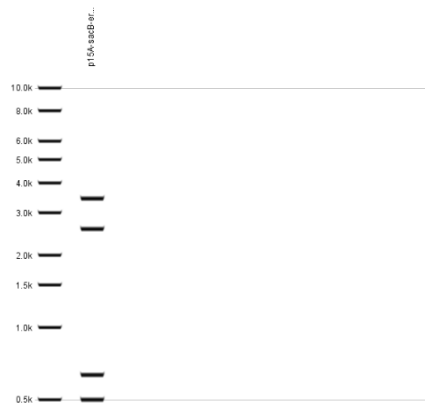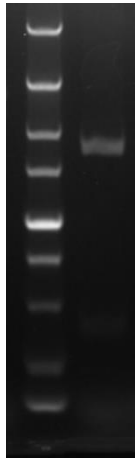

Supplement: Figure 5—figure supplement 1—source data 2. [file elife-100068-fig5-figsupp1-data2.zip › Figure 5—figure supplement 1-source data 2.pdf]

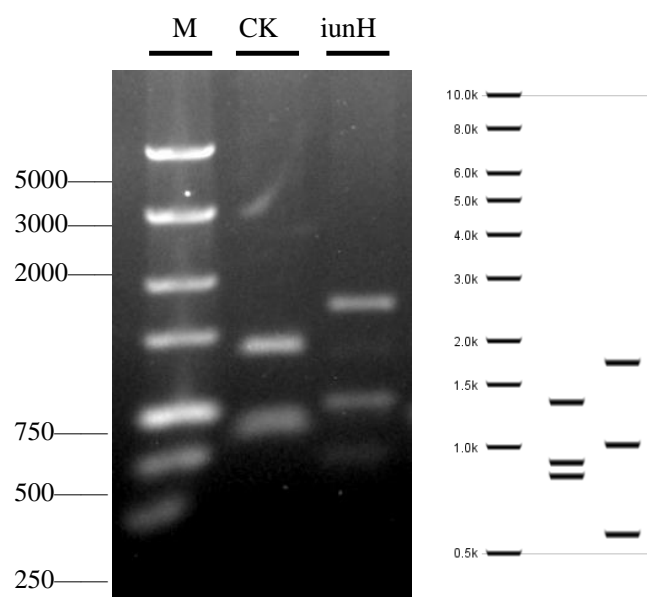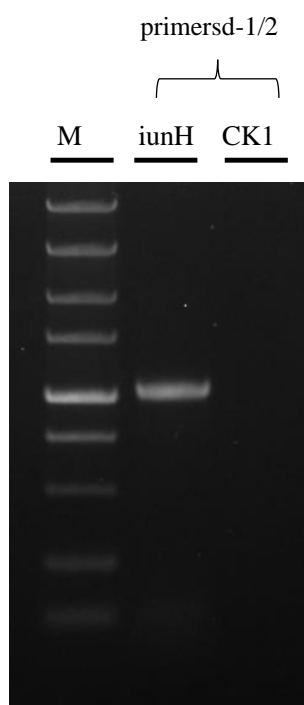

Supplement: Figure 5—figure supplement 1—source data 2. [file elife-100068-fig5-figsupp1-data2.zip › Figure 5—figure supplement 1-source data 1.pdf]

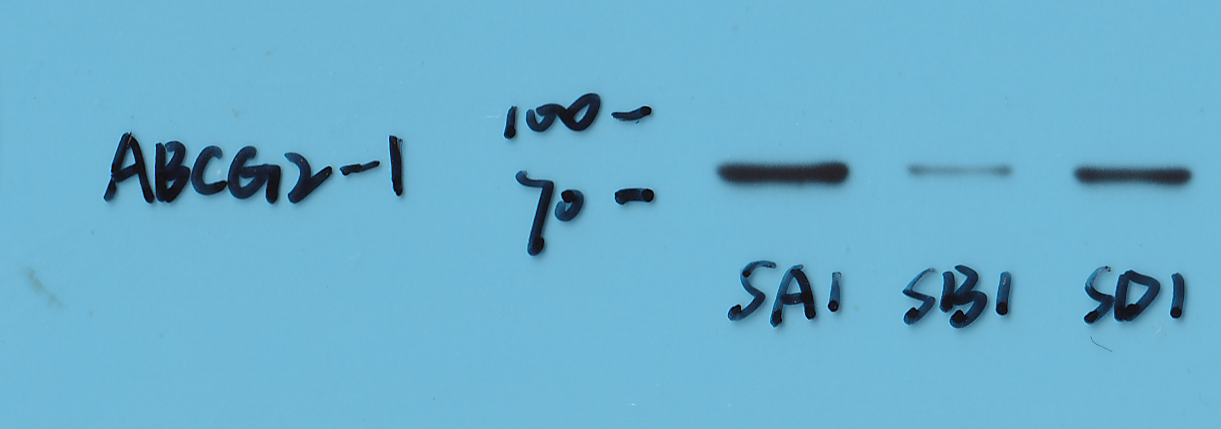

Supplement: Figure 9—source data 1. [file elife-100068-fig9-data1.zip › kidney/ABCG2-1.tif]

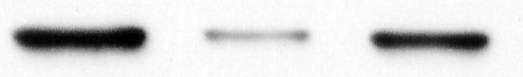

Supplement: Figure 9—source data 1. [file elife-100068-fig9-data1.zip › kidney/ABCG2-1╗╥.tif]

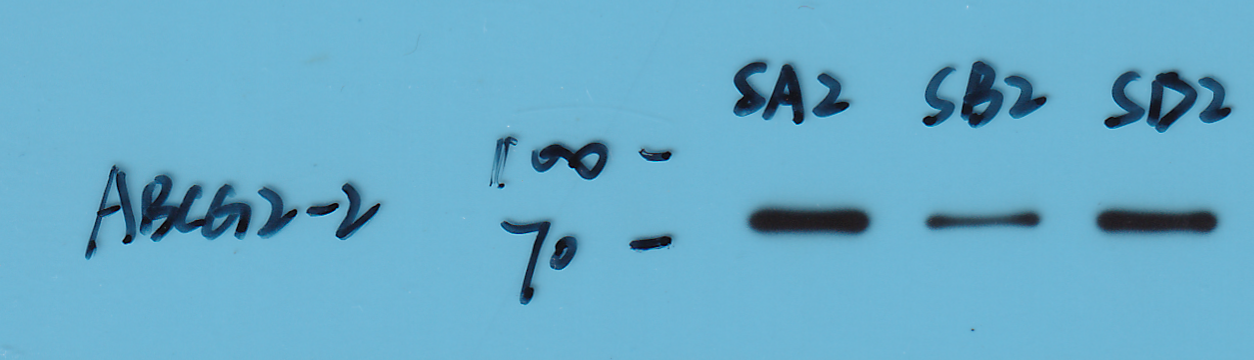

Supplement: Figure 9—source data 1. [file elife-100068-fig9-data1.zip › kidney/ABCG2-2.tif]

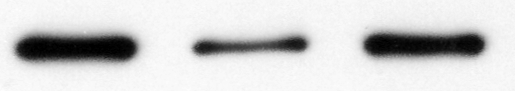

Supplement: Figure 9—source data 1. [file elife-100068-fig9-data1.zip › kidney/ABCG2-2╗╥.tif]

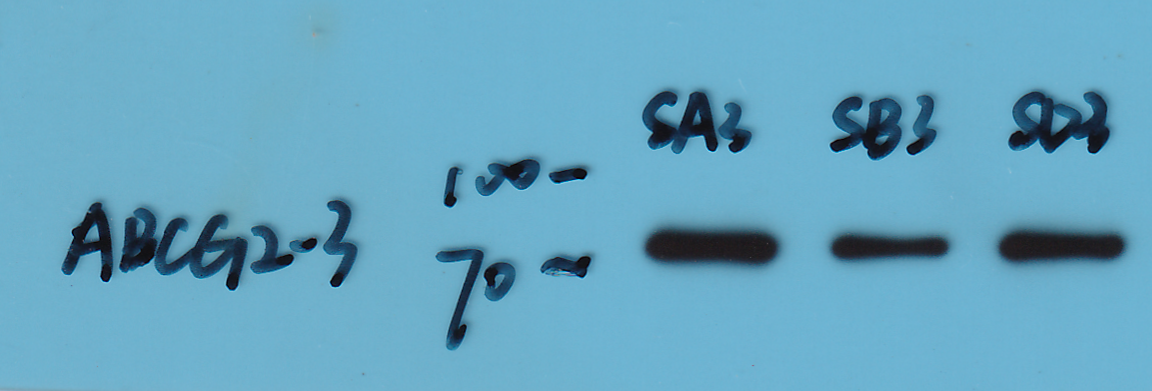

Supplement: Figure 9—source data 1. [file elife-100068-fig9-data1.zip › kidney/ABCG2-3.tif]

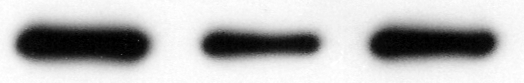

Supplement: Figure 9—source data 1. [file elife-100068-fig9-data1.zip › kidney/ABCG2-3╗╥.tif]

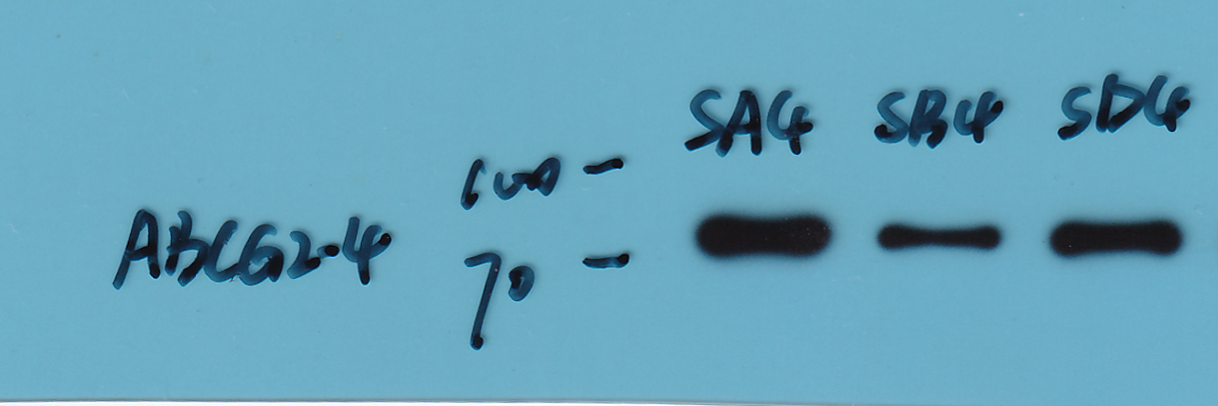

Supplement: Figure 9—source data 1. [file elife-100068-fig9-data1.zip › kidney/ABCG2-4.tif]

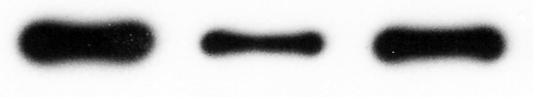

Supplement: Figure 9—source data 1. [file elife-100068-fig9-data1.zip › kidney/ABCG2-4╗╥.tif]

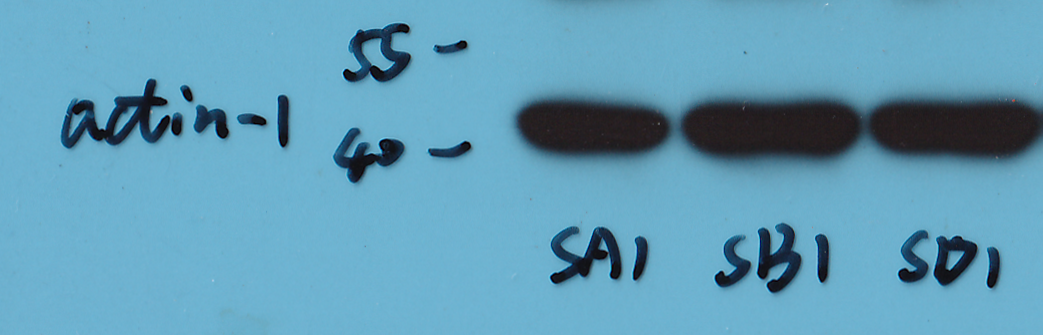

Supplement: Figure 9—source data 1. [file elife-100068-fig9-data1.zip › kidney/actin-1.tif]

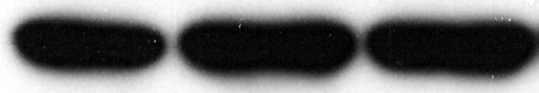

Supplement: Figure 9—source data 1. [file elife-100068-fig9-data1.zip › kidney/actin-1╗╥.tif]

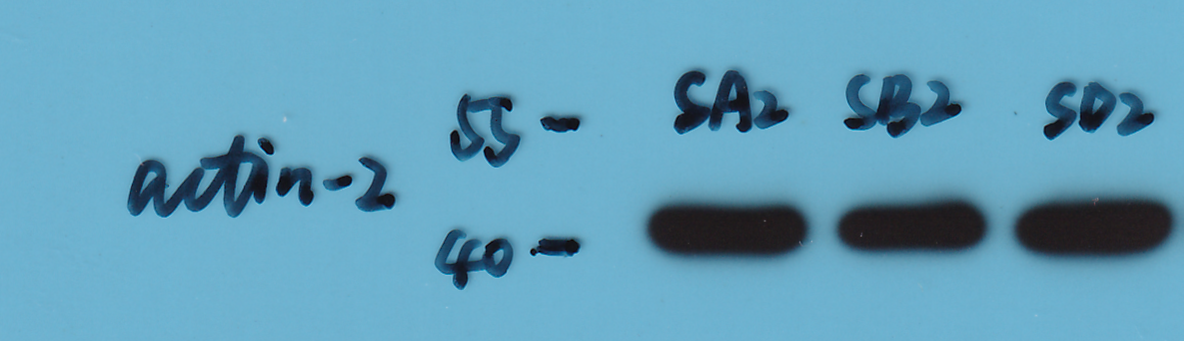

Supplement: Figure 9—source data 1. [file elife-100068-fig9-data1.zip › kidney/actin-2.tif]

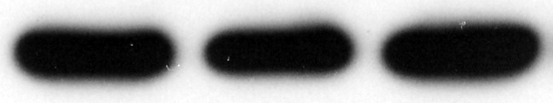

Supplement: Figure 9—source data 1. [file elife-100068-fig9-data1.zip › kidney/actin-2╗╥.tif]

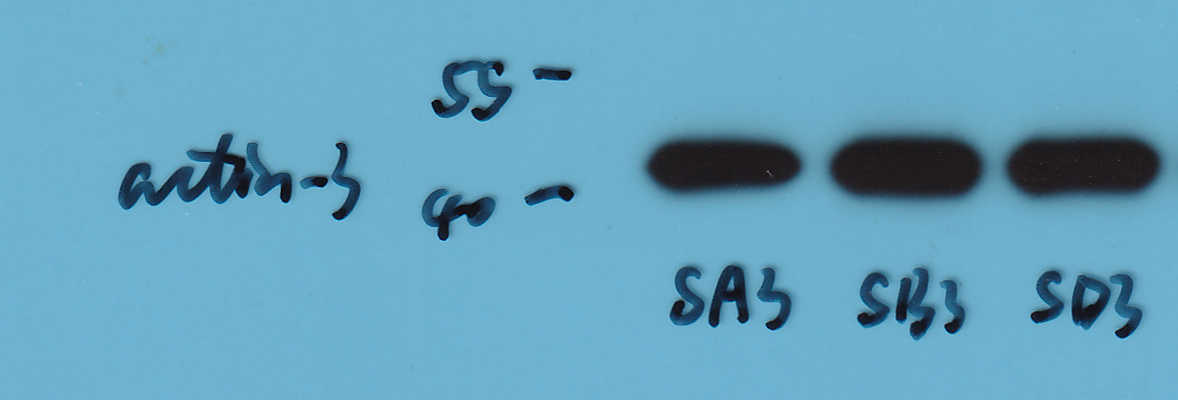

Supplement: Figure 9—source data 1. [file elife-100068-fig9-data1.zip › kidney/actin-3.tif]

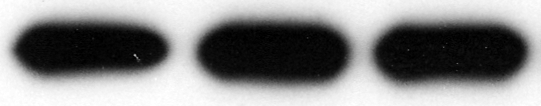

Supplement: Figure 9—source data 1. [file elife-100068-fig9-data1.zip › kidney/actin-3╗╥.tif]

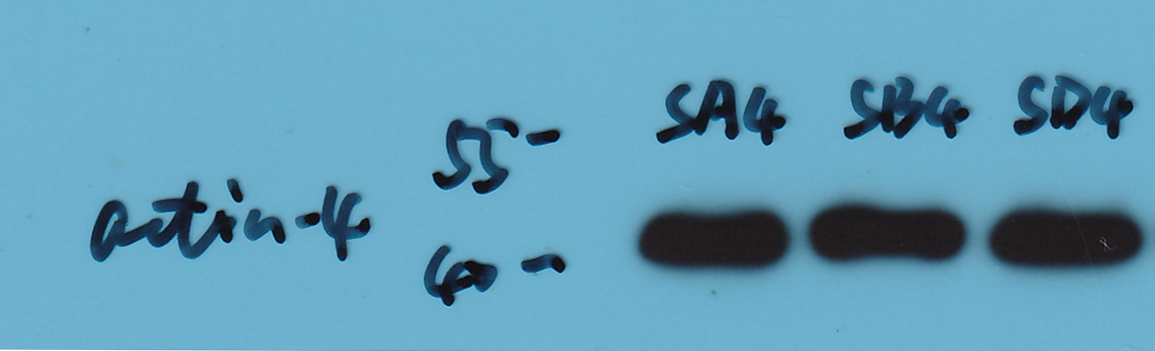

Supplement: Figure 9—source data 1. [file elife-100068-fig9-data1.zip › kidney/actin-4.tif]

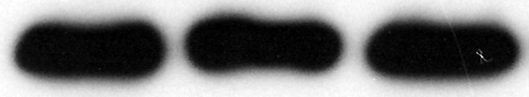

Supplement: Figure 9—source data 1. [file elife-100068-fig9-data1.zip › kidney/actin-4╗╥.tif]

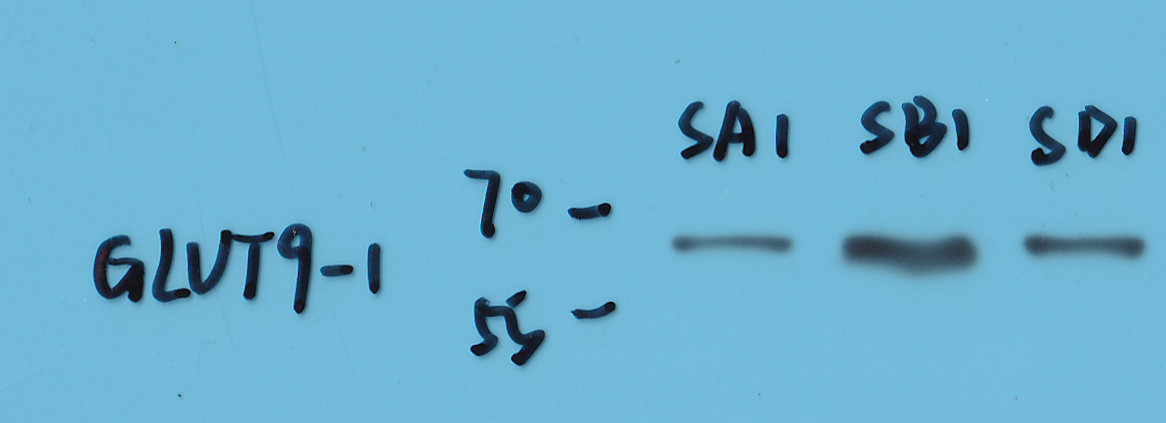

Supplement: Figure 9—source data 1. [file elife-100068-fig9-data1.zip › kidney/GLUT9-1.tif]

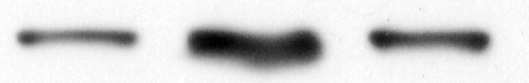

Supplement: Figure 9—source data 1. [file elife-100068-fig9-data1.zip › kidney/GLUT9-1╗╥.tif]

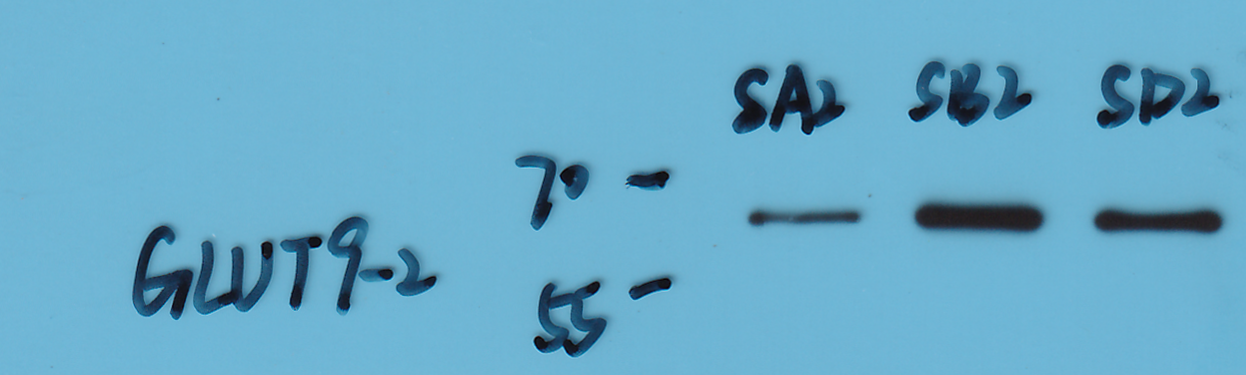

Supplement: Figure 9—source data 1. [file elife-100068-fig9-data1.zip › kidney/GLUT9-2.tif]

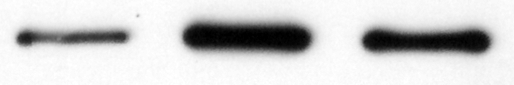

Supplement: Figure 9—source data 1. [file elife-100068-fig9-data1.zip › kidney/GLUT9-2╗╥.tif]

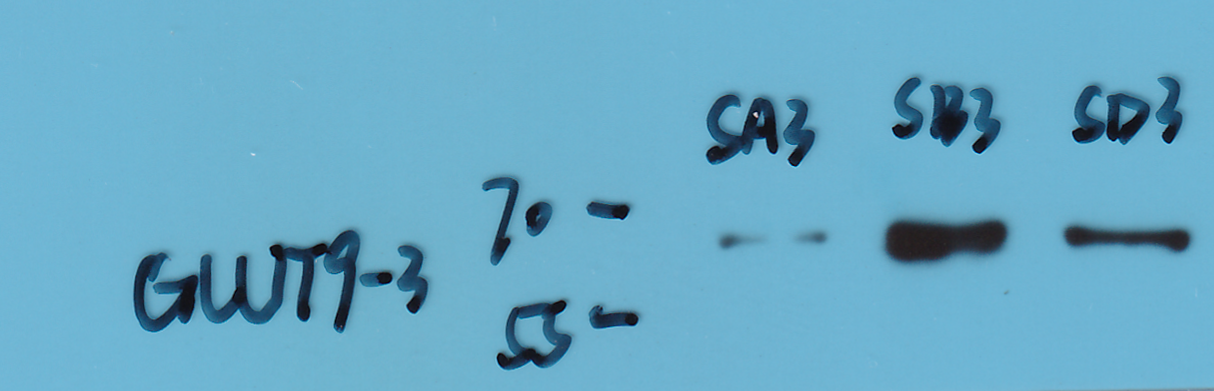

Supplement: Figure 9—source data 1. [file elife-100068-fig9-data1.zip › kidney/GLUT9-3.tif]

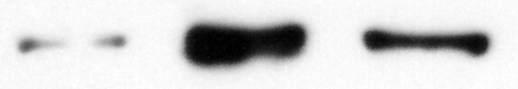

Supplement: Figure 9—source data 1. [file elife-100068-fig9-data1.zip › kidney/GLUT9-3╗╥.tif]

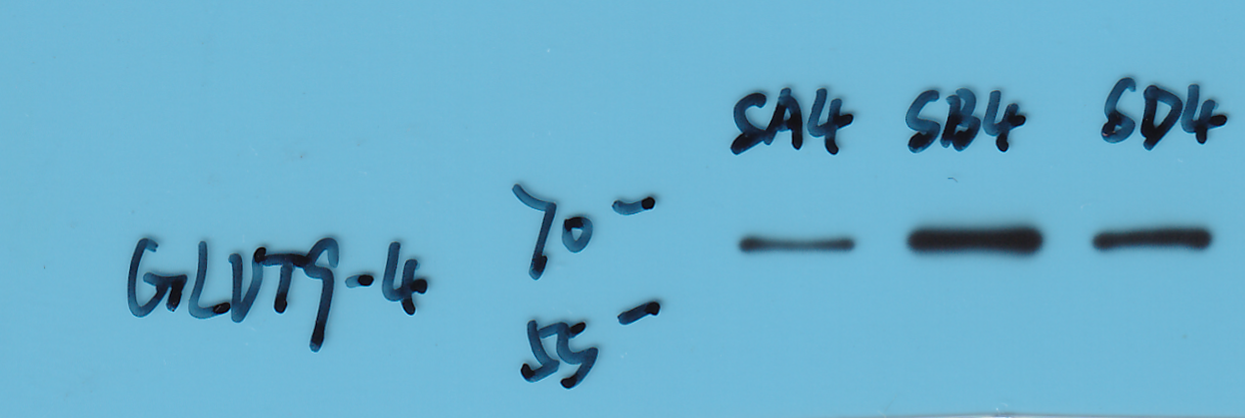

Supplement: Figure 9—source data 1. [file elife-100068-fig9-data1.zip › kidney/GLUT9-4.tif]

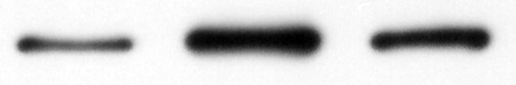

Supplement: Figure 9—source data 1. [file elife-100068-fig9-data1.zip › kidney/GLUT9-4╗╥.tif]

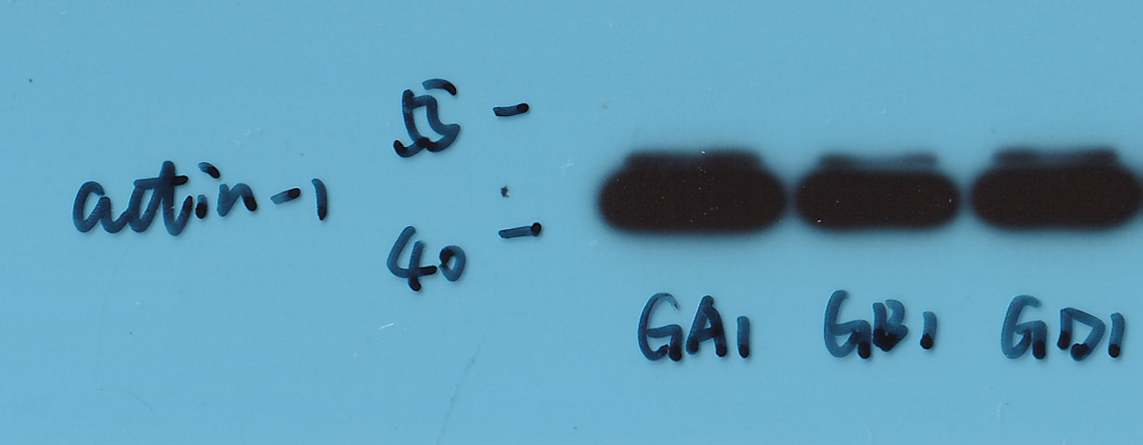

Supplement: Figure 9—source data 1. [file elife-100068-fig9-data1.zip › liver/actin-1.tif]

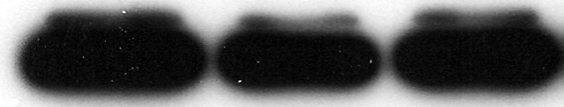

Supplement: Figure 9—source data 1. [file elife-100068-fig9-data1.zip › liver/actin-1╗╥.tif]

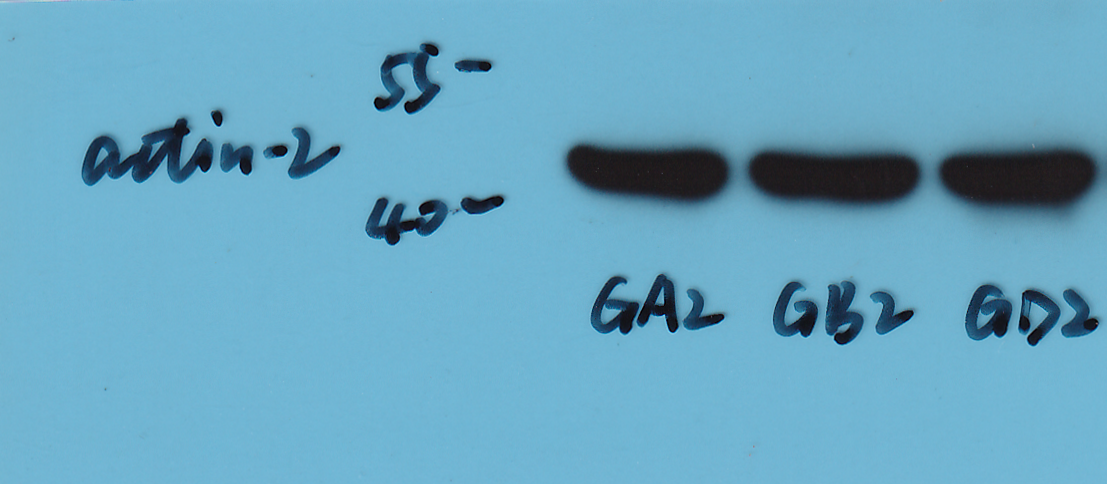

Supplement: Figure 9—source data 1. [file elife-100068-fig9-data1.zip › liver/actin-2.tif]

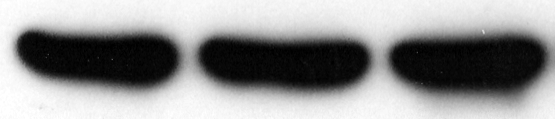

Supplement: Figure 9—source data 1. [file elife-100068-fig9-data1.zip › liver/actin-2╗╥.tif]

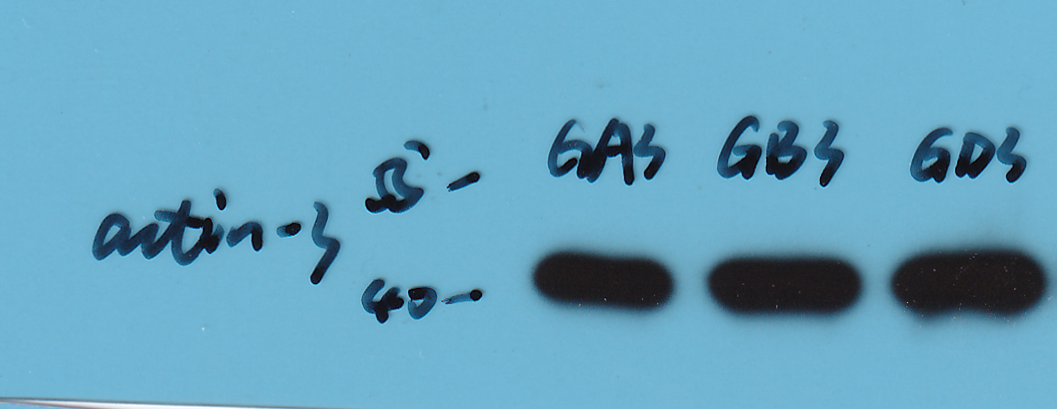

Supplement: Figure 9—source data 1. [file elife-100068-fig9-data1.zip › liver/actin-3.tif]

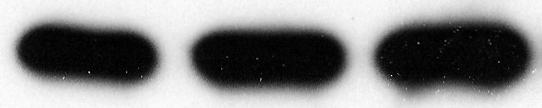

Supplement: Figure 9—source data 1. [file elife-100068-fig9-data1.zip › liver/actin-3╗╥.tif]

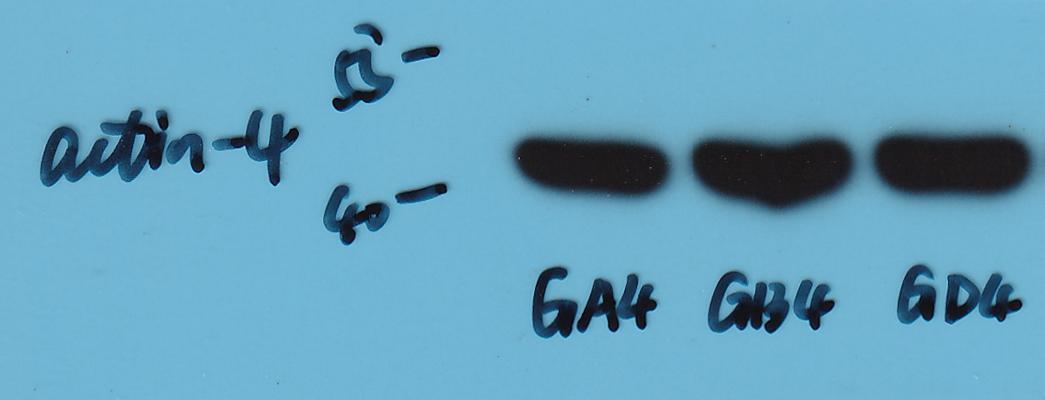

Supplement: Figure 9—source data 1. [file elife-100068-fig9-data1.zip › liver/actin-4.tif]

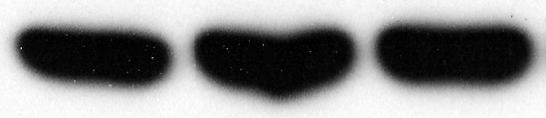

Supplement: Figure 9—source data 1. [file elife-100068-fig9-data1.zip › liver/actin-4╗╥.tif]

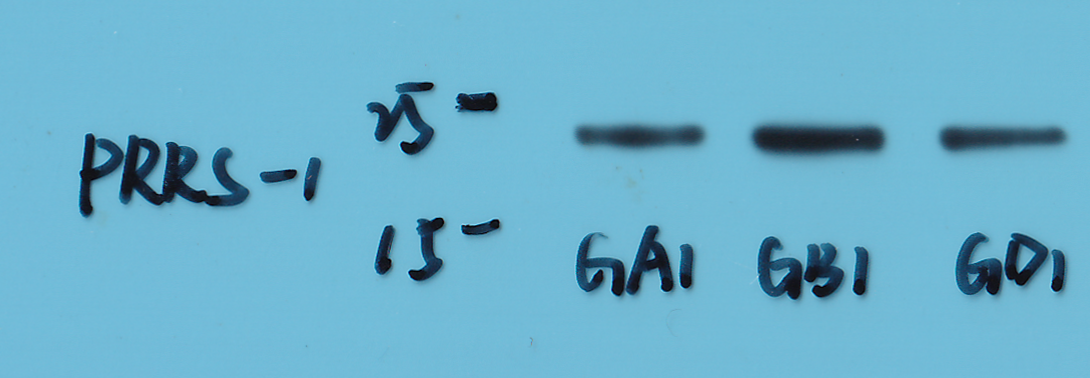

Supplement: Figure 9—source data 1. [file elife-100068-fig9-data1.zip › liver/PRRS-1.tif]

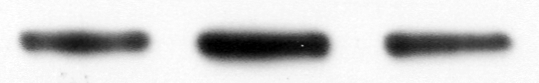

Supplement: Figure 9—source data 1. [file elife-100068-fig9-data1.zip › liver/PRRS-1╗╥.tif]

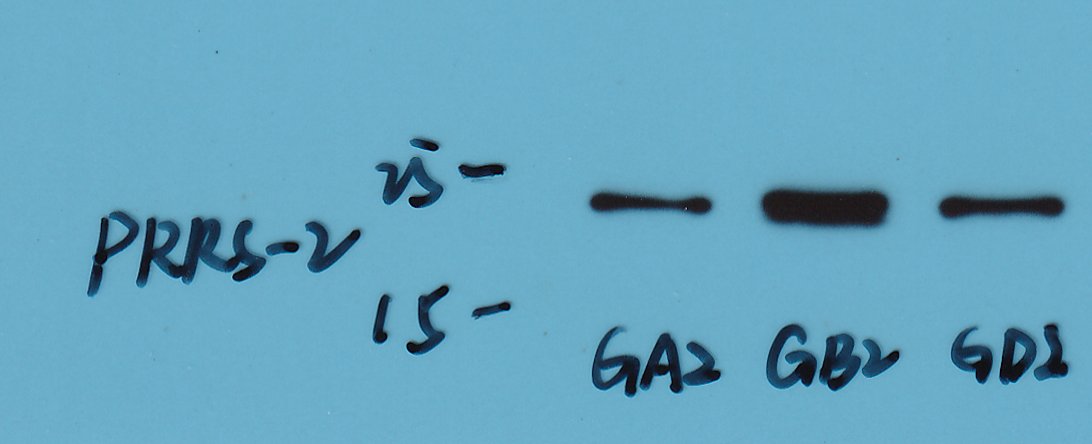

Supplement: Figure 9—source data 1. [file elife-100068-fig9-data1.zip › liver/PRRS-2.tif]

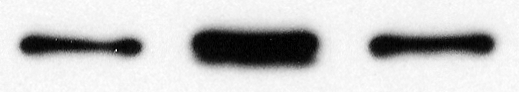

Supplement: Figure 9—source data 1. [file elife-100068-fig9-data1.zip › liver/PRRS-2╗╥.tif]

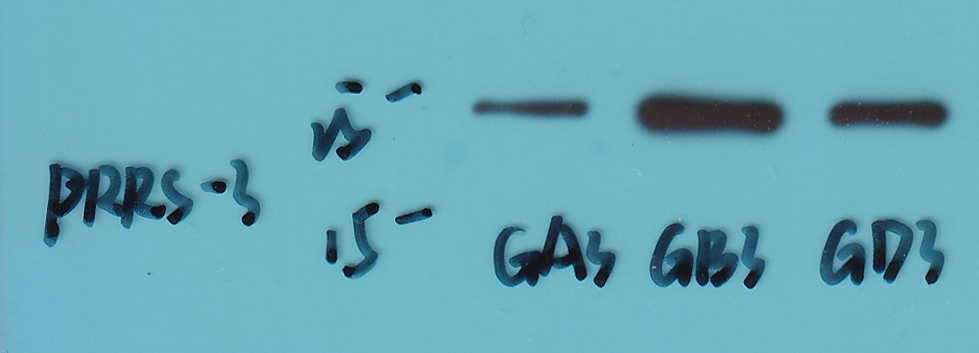

Supplement: Figure 9—source data 1. [file elife-100068-fig9-data1.zip › liver/PRRS-3.tif]

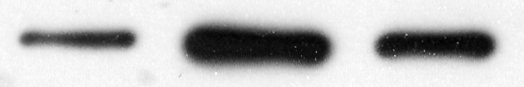

Supplement: Figure 9—source data 1. [file elife-100068-fig9-data1.zip › liver/PRRS-3╗╥.tif]

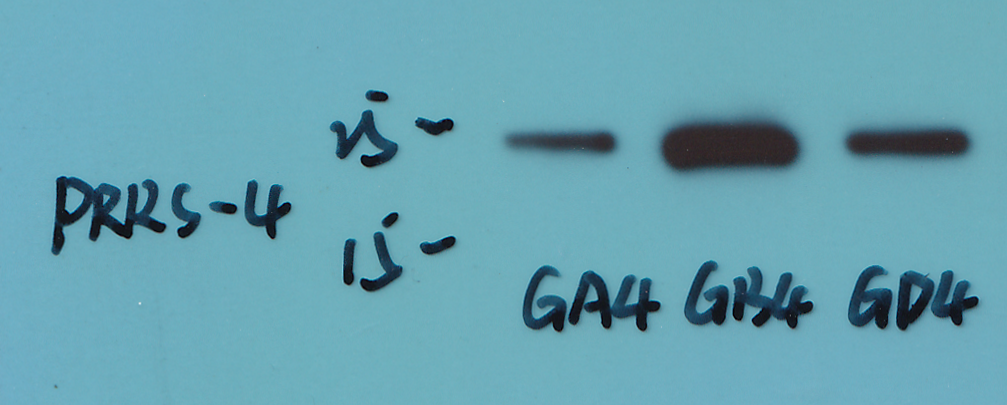

Supplement: Figure 9—source data 1. [file elife-100068-fig9-data1.zip › liver/PRRS-4.tif]

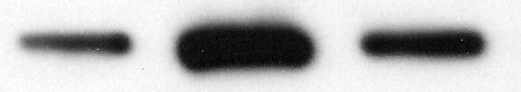

Supplement: Figure 9—source data 1. [file elife-100068-fig9-data1.zip › liver/PRRS-4╗╥.tif]

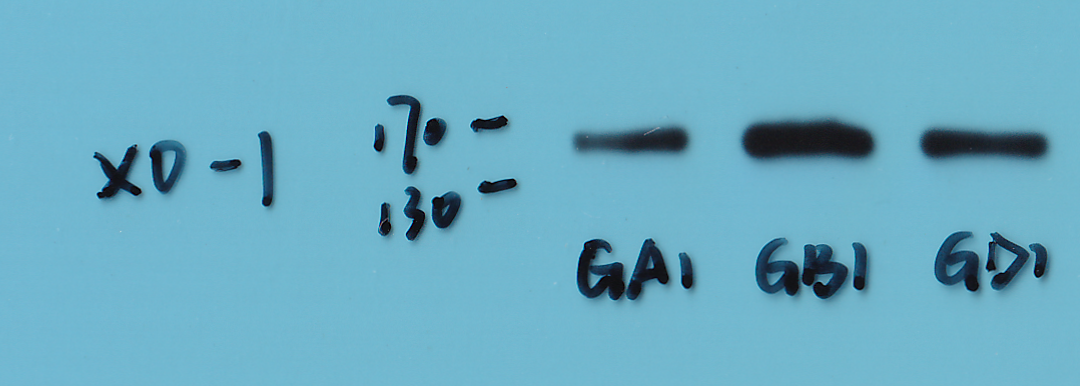

Supplement: Figure 9—source data 1. [file elife-100068-fig9-data1.zip › liver/XO-1.tif]

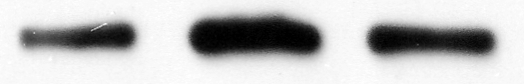

Supplement: Figure 9—source data 1. [file elife-100068-fig9-data1.zip › liver/XO-1╗╥.tif]

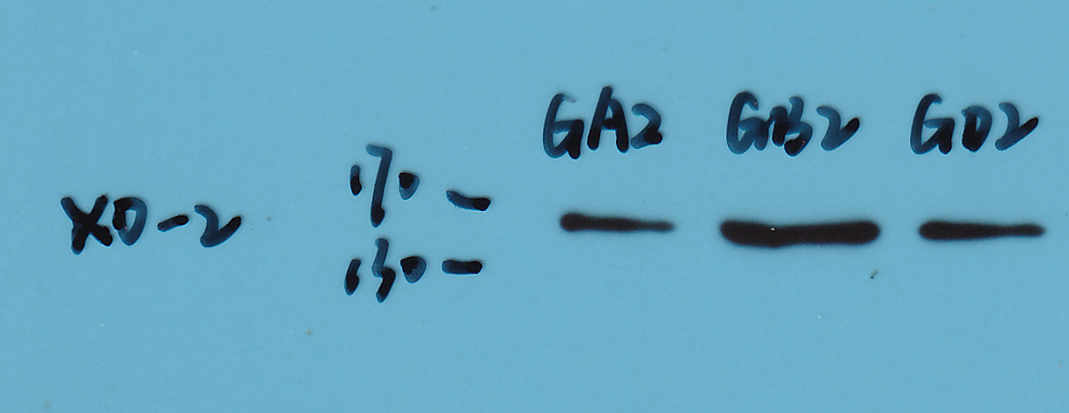

Supplement: Figure 9—source data 1. [file elife-100068-fig9-data1.zip › liver/XO-2.tif]

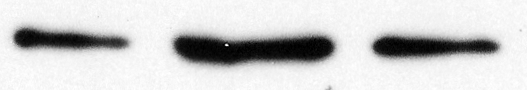

Supplement: Figure 9—source data 1. [file elife-100068-fig9-data1.zip › liver/XO-2╗╥.tif]

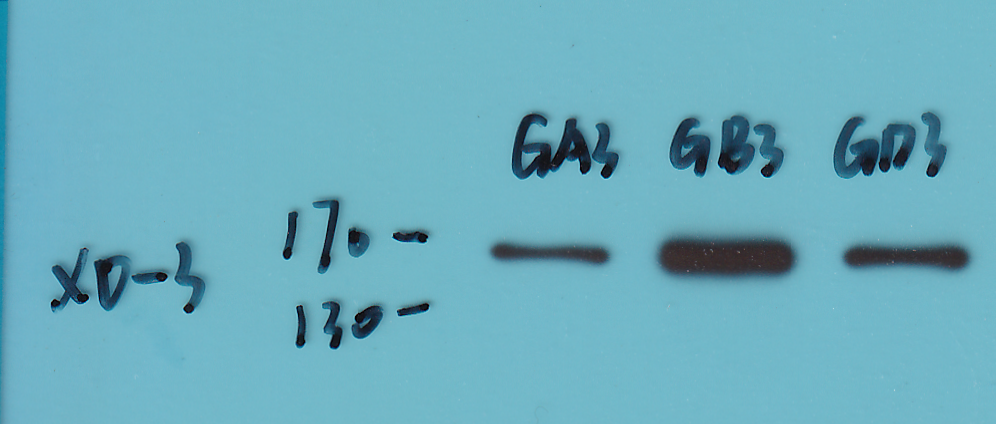

Supplement: Figure 9—source data 1. [file elife-100068-fig9-data1.zip › liver/XO-3.tif]

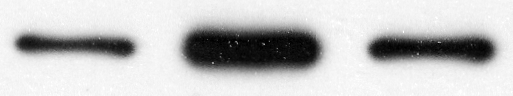

Supplement: Figure 9—source data 1. [file elife-100068-fig9-data1.zip › liver/XO-3╗╥.tif]

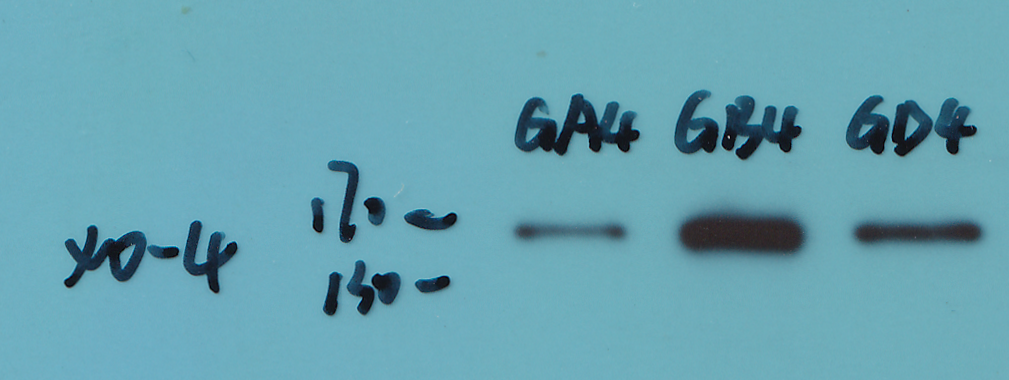

Supplement: Figure 9—source data 1. [file elife-100068-fig9-data1.zip › liver/XO-4.tif]

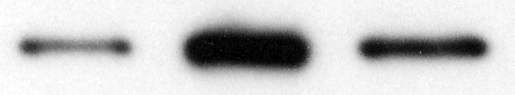

Supplement: Figure 9—source data 1. [file elife-100068-fig9-data1.zip › liver/XO-4╗╥.tif]
