# Supplementary material for: Host-derived Lactobacillus plantarum alleviates hyperuricemia by improving gut microbial community and hydrolase-mediated degradation of purine nucleosides
Source: eLife. 2024 Nov 7;13:e100068. doi: 10.7554/eLife.100068 (PMC11542919; doi:10.7554/eLife.100068)
Supplement: Figure 9—source data 2. [file elife-100068-fig9-data2.zip › Figure 9-source data 2.pdf]

Figure 9

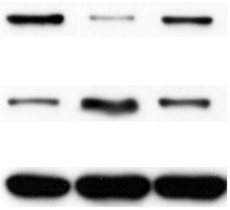

ABCG2-1

GLUT9-1

actin-1

编号	SA1	SB1	SD1
ABCG2-1	42.89	6.36	27.82
actin	115.99	143.85	138.57
ABCG2-1/actin	0.37	0.04	0.20

编号	SA1	SB1	SD1
GLUT9-1	14.27	53.88	26.37
actin	115.99	143.85	138.57
GLUT9-1/actin	0.12	0.37	0.19

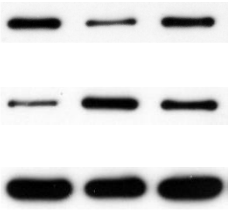

ABCG2-2

GLUT9-2

actin-2

编号	SA2	SB2	SD2
ABCG2	43.84	19.35	39.07
actin	108.48	101.76	113.85
ABCG2/actin	0.40	0.19	0.34

编号	SA2	SB2	SD2
GLUT9	15.92	50.44	39.37
actin	108.48	101.76	113.85
GLUT9/actin	0.15	0.50	0.35

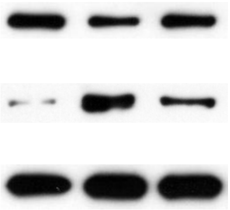

ABCG2-3

GLUT9-3

actin-3

编号	SA3	SB3	SD3
ABCG2	64.30	34.65	54.65
actin	100.61	120.12	115.77
ABCG2/actin	0.64	0.29	0.47

编号	SA3	SB3	SD3
GLUT9	6.58	62.88	30.01
actin	100.61	120.12	115.77
GLUT9/actin	0.07	0.52	0.26

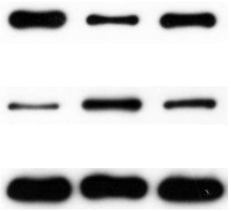

ABCG2-4

GLUT9-4

actin-4

编号	SA4	SB4	SD4
ABCG2	71.88	33.58	57.47
actin	117.71	117.21	118.84
ABCG2/actin	0.61	0.29	0.48

编号	SA4	SB4	SD4
GLUT9	14.95	49.15	31.86
actin	117.71	117.21	118.84
GLUT9/actin	0.13	0.42	0.27

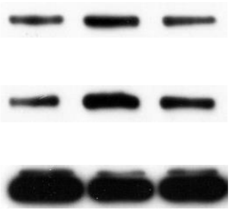

PRRS-1

XO-1

actin-1

编号	GA1	GB1	GD1
PRRS	23.71	41.33	23.57
actin	125.14	104.38	116.76
PRRS/actin	0.19	0.40	0.20

编号	GA1	GB1	GD1
XO	23.86	56.70	37.95
actin	125.14	104.38	116.76
XO/actin	0.19	0.54	0.33

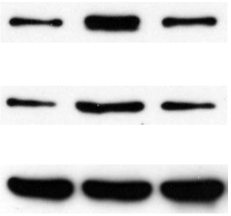

PRRS-2

XO-2

actin-2

编号	GA2	GB2	GD2
PRRS	21.97	58.14	29.48
actin	95.49	101.30	104.22
PRRS/actin	0.23	0.57	0.28

编号	GA2	GB2	GD2
XO	16.98	47.66	25.58
actin	95.49	101.30	104.22
XO/actin	0.18	0.47	0.25

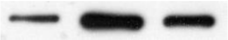

PRRS-3

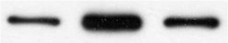

XO-3

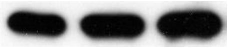

actin-3

编号	GA3	GB3	GD3
PRRS	15.36	61.49	35.51
actin	96.84	111.00	120.55
PRRS/actin	0.16	0.55	0.29

编号	GA3	GB3	GD3
XO	18.79	62.61	32.05
actin	96.84	111.00	120.55
XO/actin	0.19	0.56	0.27

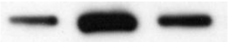

PRRS-4

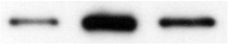

XO-4

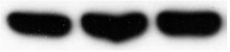

actin-4

编号	GA4	GB4	GD4
PRRS	18.41	76.86	38.88
actin	102.31	117.48	112.96
PRRS/actin	0.18	0.65	0.34

编号	GA4	GB4	GD4
XO	10.36	61.06	28.26
actin	102.31	117.48	112.96
XO/actin	0.10	0.52	0.25
