# Supplementary material for: Host-derived Lactobacillus plantarum alleviates hyperuricemia by improving gut microbial community and hydrolase-mediated degradation of purine nucleosides
Source: eLife. 2024 Nov 7;13:e100068. doi: 10.7554/eLife.100068 (PMC11542919; doi:10.7554/eLife.100068)
Supplement: Figure 9—source data 2. [file elife-100068-fig9-data2.zip › Figure 9-source data 2.docx]

Figure 9


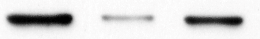
 ABCG2-1


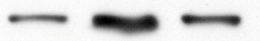
 GLUT9-1


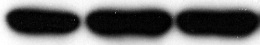
 actin-1

| 编号 | SA1 | SB1 | SD1 |
| --- | --- | --- | --- |
| ABCG2-1 | 42.89 | 6.36 | 27.82 |
| actin | 115.99 | 143.85 | 138.57 |
| ABCG2-1/actin | 0.37 | 0.04 | 0.20 |

| 编号 | SA1 | SB1 | SD1 |
| --- | --- | --- | --- |
| GLUT9-1 | 14.27 | 53.88 | 26.37 |
| actin | 115.99 | 143.85 | 138.57 |
| GLUT9-1/actin | 0.12 | 0.37 | 0.19 |


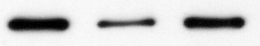
 ABCG2-2


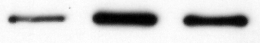
 GLUT9-2


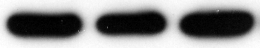
 actin-2

| 编号 | SA2 | SB2 | SD2 |
| --- | --- | --- | --- |
| ABCG2 | 43.84 | 19.35 | 39.07 |
| actin | 108.48 | 101.76 | 113.85 |
| ABCG2/actin | 0.40 | 0.19 | 0.34 |

| 编号 | SA2 | SB2 | SD2 |
| --- | --- | --- | --- |
| GLUT9 | 15.92 | 50.44 | 39.37 |
| actin | 108.48 | 101.76 | 113.85 |
| GLUT9/actin | 0.15 | 0.50 | 0.35 |


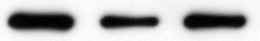
 ABCG2-3


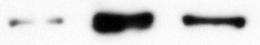
 GLUT9-3


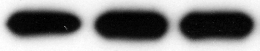
 actin-3

| 编号 | SA3 | SB3 | SD3 |
| --- | --- | --- | --- |
| ABCG2 | 64.30 | 34.65 | 54.65 |
| actin | 100.61 | 120.12 | 115.77 |
| ABCG2/actin | 0.64 | 0.29 | 0.47 |

| 编号 | SA3 | SB3 | SD3 |
| --- | --- | --- | --- |
| GLUT9 | 6.58 | 62.88 | 30.01 |
| actin | 100.61 | 120.12 | 115.77 |
| GLUT9/actin | 0.07 | 0.52 | 0.26 |


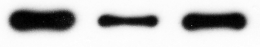
 ABCG2-4


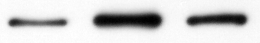
 GLUT9-4


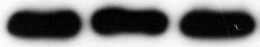
 actin-4

| 编号 | SA4 | SB4 | SD4 |
| --- | --- | --- | --- |
| ABCG2 | 71.88 | 33.58 | 57.47 |
| actin | 117.71 | 117.21 | 118.84 |
| ABCG2/actin | 0.61 | 0.29 | 0.48 |

| 编号 | SA4 | SB4 | SD4 |
| --- | --- | --- | --- |
| GLUT9 | 14.95 | 49.15 | 31.86 |
| actin | 117.71 | 117.21 | 118.84 |
| GLUT9/actin | 0.13 | 0.42 | 0.27 |


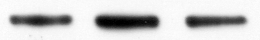
 PRRS-1


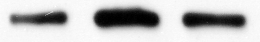
 XO-1


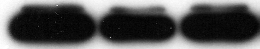
 actin-1

| 编号 | GA1 | GB1 | GD1 |
| --- | --- | --- | --- |
| PRRS | 23.71 | 41.33 | 23.57 |
| actin | 125.14 | 104.38 | 116.76 |
| PRRS/actin | 0.19 | 0.40 | 0.20 |

| 编号 | GA1 | GB1 | GD1 |
| --- | --- | --- | --- |
| XO | 23.86 | 56.70 | 37.95 |
| actin | 125.14 | 104.38 | 116.76 |
| XO/actin | 0.19 | 0.54 | 0.33 |


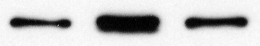
 PRRS-2


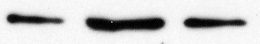
 XO-2


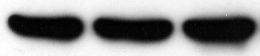
 actin-2

| 编号 | GA2 | GB2 | GD2 |
| --- | --- | --- | --- |
| PRRS | 21.97 | 58.14 | 29.48 |
| actin | 95.49 | 101.30 | 104.22 |
| PRRS/actin | 0.23 | 0.57 | 0.28 |

| 编号 | GA2 | GB2 | GD2 |
| --- | --- | --- | --- |
| XO | 16.98 | 47.66 | 25.58 |
| actin | 95.49 | 101.30 | 104.22 |
| XO/actin | 0.18 | 0.47 | 0.25 |


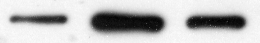
 PRRS-3


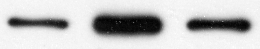
 XO-3


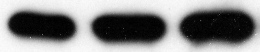
 actin-3

| 编号 | GA3 | GB3 | GD3 |
| --- | --- | --- | --- |
| PRRS | 15.36 | 61.49 | 35.51 |
| actin | 96.84 | 111.00 | 120.55 |
| PRRS/actin | 0.16 | 0.55 | 0.29 |

| 编号 | GA3 | GB3 | GD3 |
| --- | --- | --- | --- |
| XO | 18.79 | 62.61 | 32.05 |
| actin | 96.84 | 111.00 | 120.55 |
| XO/actin | 0.19 | 0.56 | 0.27 |


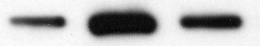
 PRRS-4


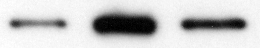
 XO-4


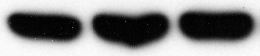
 actin-4

| 编号 | GA4 | GB4 | GD4 |
| --- | --- | --- | --- |
| PRRS | 18.41 | 76.86 | 38.88 |
| actin | 102.31 | 117.48 | 112.96 |
| PRRS/actin | 0.18 | 0.65 | 0.34 |

| 编号 | GA4 | GB4 | GD4 |
| --- | --- | --- | --- |
| XO | 10.36 | 61.06 | 28.26 |
| actin | 102.31 | 117.48 | 112.96 |
| XO/actin | 0.10 | 0.52 | 0.25 |
