# Supplementary material for: Host-derived Lactobacillus plantarum alleviates hyperuricemia by improving gut microbial community and hydrolase-mediated degradation of purine nucleosides
Source: eLife. 2024 Nov 7;13:e100068. doi: 10.7554/eLife.100068 (PMC11542919; doi:10.7554/eLife.100068)
Supplement: Supplementary file 1. [file elife-100068-supp1.docx]

**Table S1. Strains, plasmids, mutants, primers, and antibiotics used in this study.**

| **Strains** | | **Description** | | **Source** |  |  |
| --- | --- | --- | --- | --- | --- | --- |
| *Lactobacillus*  *_plantarum* | |  | | Lab stock |  |  |
| *E. coli GB2005* | | (HS996, ∆recET, ∆ybcC). The endogenous recET locus and the DLP12 prophage ybcC, which encodes a putative exonuclease similar to the Redα, were deleted | | Lab stock |  |  |
| *E. coli GB05-dir* | | (GB2005, araC-BAD-ETgA) recE, recT, redγ and recA under BAD promoter was inserted at the ybcC locus | | Lab stock |  |  |
| *Nissle1917/p15A-cm-Pgenta* | | The Pgenta promoter gene was heterologous expressed in E. coli Nissle1917 | | This study |  |  |
| *Nissle1917/p15A-cm-iunH* | | The iunH gene was heterologous expressed in E. coli Nissle1917 | | This study |  |  |
| **Plasmids** | | **Characteristics** | | **Source** |  |  |
| pBBR1-Rha-redγβα-kan | | pBBR1 replicon, kmR, redγβα under the control of Rha promoter | | Lab stock |  |  |
| p15A-cm-tetR-tetO-hyg-ccdB | | p15A replicon, c*mR* | | Lab stock |  |  |
| pMSP3535 | | pBR322 replicon, ermR, repE, nisR, nisK, under the control of T7 promoter | | Lab stock |  |  |
| RK2-pBAD-Cre-Dre-sacB | | pBAD replicon, apraR, kmR | | Lab stock |  |  |
| pNBU2-erm-genta-cspRecT | | pNBU2 replicon, gentaR | | Lab stock |  |  |
| pBBR1-Rha-redγβα-Kan-erm | | pBBR1 replicon, km^R^, redγβα under the control of Rha promoter | | Lab stock |  |  |
| p15A-cm-Pgenta | | p15A replicon, cm^R^, iunH under the control of Pgenta promoter | | This study |  |  |
| p15A-cm-iunH | | p15A replicon, cm^R^, iunH under the control of Pgenta promoter | | This study |  |  |
| p15A-cm-HA-erm-scaB-iunH | | p15A replicon, cm^R^, scaB under the control of erm promoter | | This study |  |  |
| **Mutants** | | **Characteristics** | | **Source** |  |  |
| LP∆iunH | | The deletion of 957 bp (2381344-2382300) region on L. plantarum chromosome was replaced by a linearized fragment of a plasmid of p15A-cm-HA-erm-scaB-iunH | | This study |  |  |
| **Primers** | | **Sequence** | | **Application** |  |  |
| p15A-5 | | AAACTACCGCATTAAAGCTT | | For amplification of p15A-cm supporter |  |  |
| p15A-3 | | CTGAACCGACGACCGGGTCG | |  |  |  |
| p15A-Pgenta-5 | | CAGAAATTCGAAAGCAAATTCGACCCGGTCGTCGGTTCAGGAAGGCACGAACCCAGTTG | | For amplification of Pgenta promotor |  |  |
| genta-60kDa-3 | | CGTTGCTGCTCCATAACAT | |  |  |  |
| Pgenta-iunH-5 | | TACGCCGTGGGTCGATGTTTGATGTTATGGAGCAGCAACGGAGGATGTTTTACATGGCA | | For amplification of iunH gene |  |  |
| iunH-3 | | TTTGACAGCTTATCATCGATAAGCTTTAATGCGGTAGTTTCTAATGGGCTTTAAATAAT | |  |  |  |
| erm-5 | | GTTCTATGCTTTCTTTTTGTAGCCGGCTAAACGGATAGTCCCCCAAAATCATCTTGCCTTTGATATTGAGGTATCATTT | | For amplification of erm resistance gen |  |  |
| erm-3 | | CCTTTTTAATCACAATTCAGAAAATATCATAATATCTCATTTCACTAAATAATAGTGAACTTAGCCGTTAAATATTTTA | |  |  |  |
| sacB-5 | | GTTCACTATTATTTAGTGAA | | For amplification of sacB gene |  |  |
| sacB-3 | | AGTGTGACTCTAGTAGAGAGCGTTCACCGACAAACAACAGTTTGTTAACTGTTAATTGT | |  |  |  |
| Pi-HAR-2 | | GACATTCCCGGCAATCGGAC | | For amplification ofp15A-cm-HA-erm-scaB-iunH plasmids |  |  |
| Pi-HAR-1 | | AAGGCAAGATGATTTTGGGG | |  |  |  |
| Pi-HAL-2 | | TTTGAACACTCATGTTTAAC | |  |  |  |
| Pi-HAL-1 | | GATGGATGTTCAACTAATTTGTCCGATTGCCGGGAATGTCGCTGATTGATCTGAAAGGA | |  |  |  |
| P15A-PiHAR-2 | | TGTTAGTCATTTTCTTTCTCAACCTCGTCATTGGCACCAAGTTAAACATGAGTGTTCAAAGACGTCGATATCTGGCGAA | |  |  |  |
| check-HA-PiunH-1 | | TTCGCTTGAGGTACAGCGAA | | For the first step of single-exchange recombinant colony PCR detection |  |  |
| check-HA-PiunH-2 | | GCTGTTAATGGCAGAGGTAG | |  |  |  |
| **Antibiotic** | | **Source** | | **Identifier** | | |
| GLUT9 (1:1000) | | Proteintech | | 26486-1-AP | | |
| ABCG2 (1:5000) | | Abcam | | ab108312 | | |
| PRRS (1:1000) | | Bioss | | bs-4504R | | |
| XO (1:5000) | | Abcam | | ab109235 | | |
| β-actin (1:5000) | | Proteintech | | 60009-1-Ig | | |

**Table S1. Strains, plasmids, mutants, primers, and antibiotics used in this study.**

The description and source for strains, plasmids, mutants, primers, and antibiotics used in this study.
